# Intraoperative High-Volume Diuresis During Off-Pump Coronary Artery Bypass Grafting: Risk Factors and Clinical Impact

**DOI:** 10.3390/jcm15062331

**Published:** 2026-03-18

**Authors:** Yuxi Hou, Fangyi Luo, Shuwen Li, Fei Cai, Jun Ma

**Affiliations:** Beijing Anzhen Hospital, Capital Medical University, Beijing 100029, China; houyuxi1013@sina.com (Y.H.); fangyi1346@163.com (F.L.); dotocrshuwen@163.com (S.L.); 13051809977@163.com (F.C.)

**Keywords:** off-pump coronary artery bypass grafting, Intraoperative high-volume diuresis, hemodynamic exposure, perioperative fluid management, renal function

## Abstract

**Background:** Intraoperative high-volume diuresis is a common but under-recognized phenomenon during off-pump coronary artery bypass grafting (OPCABG). Its clinical correlates and implications for perioperative management remain incompletely characterized. **Methods:** This single-center retrospective cohort study included 1274 adults undergoing elective OPCABG between January and August 2025. High-volume diuresis was defined as urine output ≥ 5 mL·kg^−1^·h^−1^. Multivariable logistic regression was used to identify factors independently associated with intraoperative high-volume diuresis. Model discrimination was assessed using the area under the receiver operating characteristic curve (AUC). **Results**: High-volume diuresis occurred in 39.6% of patients. Older age, hypertension and greater intraoperative fluid infusion were independently associated with high-volume diuresis, whereas preoperative diuretic and greater cumulative exposure to systolic blood pressure < 100 mmHg were inversely associated with diuresis. The multivariable model demonstrated acceptable discrimination (AUC = 0.756). Postoperative outcomes, including acute kidney injury, duration of mechanical ventilation, intensive care unit stay, and hospital length of stay, did not differ between groups. **Conclusions**: Intraoperative high-volume diuresis during OPCABG reflects complex physiological and hemodynamic responses and can be anticipated based on preoperative and intraoperative factors. These findings support a more individualized interpretation of urine output and perioperative management strategies in OPCABG.

## 1. Introduction

Intraoperative high-volume diuresis is a frequently observed yet insufficiently studied phenomenon in perioperative practice. Excessive urine output during major surgery may lead to rapid reductions in effective circulating blood volume, provoke hemodynamic instability, and contribute to electrolyte disturbances, collectively posing challenges for maintaining adequate organ perfusion and postoperative recovery. Although cases have been reported in thoracic, spinal, and neurosurgical procedures, the mechanisms driving intraoperative high-volume diuresis remain poorly defined, and its clinical significance is uncertain [1,2,3].

Off-pump coronary artery bypass grafting (OPCABG) presents a unique physiological environment in which the heart is repeatedly displaced to achieve surgical exposure, often resulting in reduced venous return, fluctuating cardiac output, and unstable hemodynamics [4,5,6]. These factors complicate perioperative fluid management, potentially influencing renal perfusion, neuroendocrine responses, and urine output regulation. Our previous work suggested that intraoperative diuresis during OPCABG may involve both osmotic factors and impaired antidiuretic signaling, indicating a multifactorial physiological process rather than a single mechanism [7].

Despite these observations, major gaps remain. The perioperative clinical factors associated with high-volume diuresis during OPCABG have not been systematically evaluated in a large cohort, and the extent to which this phenomenon affects postoperative outcomes is unknown. A clearer understanding is essential, as excessive intraoperative urine output frequently prompts reactive adjustments in fluid and vasoactive therapy and may influence perioperative decision-making.

Therefore, the present study aimed to identify perioperative risk factors associated with intraoperative high-volume diuresis in patients undergoing OPCABG and to assess its potential impact on perioperative and short-term postoperative outcomes. By clarifying both the determinants and clinical relevance of this phenomenon, we seek to support more individualized and physiologically informed perioperative management strategies.

## 2. Materials and Methods

This was a single-center retrospective cohort study. Adult patients who underwent off-pump coronary artery bypass grafting (OPCABG) at Beijing Anzhen Hospital Tongzhou Campus, Capital Medical University, between January 2025 and August 2025 were consecutively enrolled. All procedures were performed under general anesthesia.

The study protocol was approved by the Ethics Committee of Beijing Anzhen Hospital (approval no. KS2025058). Given the retrospective design of the study, the requirement for informed consent was waived by the ethics committee.

### 2.1. Participants

Adult patients aged 18–75 years who underwent elective off-pump coronary artery bypass grafting were consecutively included, provided that complete intraoperative urine output data and hemodynamic monitoring records were available.

Patients were excluded if they had undergone preoperative renal replacement therapy, had moderate-to-severe pre-existing renal dysfunction (defined as an estimated glomerular filtration rate [eGFR] < 60 mL/min/1.73 m^2^), required conversion from off-pump to cardiopulmonary bypass during surgery, or had substantial missing perioperative data that precluded meaningful analysis. Patients requiring perioperative mechanical circulatory support, including intra-aortic balloon pump (IABP) or extracorporeal membrane oxygenation (ECMO), were also excluded. These exclusion criteria were applied to minimize the confounding influence of severe circulatory instability and to ensure that the observed associations primarily reflected typical perioperative physiological responses during OPCABG.

### 2.2. Intraoperative Management and Data Collection

All patients received anesthesia and perioperative management according to the standardized protocols of our institution. Anesthesia was induced with remimazolam, etomidate, rocuronium, and sufentanil, and maintained with propofol, dexmedetomidine, sufentanil, and rocuronium. Vasoactive agents such as dopamine and norepinephrine were administered intraoperatively at the discretion of the attending anesthesiologist in response to hemodynamic changes.

Perioperative data were retrospectively extracted from the electronic medical record system and the anesthesia information management system. Variables collected included demographic characteristics, comorbidities, preoperative laboratory values, intraoperative hemodynamic measurements, fluid administration details, medication use, and postoperative outcomes.

Intraoperative hemodynamic parameters, including blood pressure and heart rate, were continuously monitored and recorded at predefined intervals. Fluid management variables encompassed total intraoperative fluid volume, crystalloid administration, and estimated blood loss. Intraoperative use of vasoactive agents and diuretics was also documented. Blood samples were obtained at standardized time points: T0 (preoperative baseline), T1 (end of surgery), T2 (48 h postoperatively), and T3 (7 days postoperatively).

### 2.3. Definition of Intraoperative High-Volume Diuresis and AKI

According to previous studies, intraoperative urine output was continuously monitored and recorded via an indwelling urinary catheter. Based on the calculated intraoperative urine output normalized to body weight and surgical duration, patients were classified into the diuresis group and the non-diuresis group. Intraoperative high-volume diuresis was defined as an intraoperative urine output ≥ 5 mL·kg^−1^·h^−1^ [7], whereas values below this threshold were classified as normal intraoperative urine output.

Postoperative acute kidney injury (AKI) was defined in accordance with the Kidney Disease: Improving Global Outcomes (KDIGO) criteria as stage 1 or higher, determined by a postoperative increase in serum creatinine of at least 1.5-fold from baseline within 7 days.

### 2.4. Hemodynamic Exposure (Blood Pressure Area Under the Curve, BP_AUC)

To quantitatively assess the impact of intraoperative blood pressure abnormalities on intraoperative high-volume diuresis, cumulative hemodynamic exposure was quantified using the blood pressure area under the curve (BP_AUC) method, which integrates both the magnitude and duration of blood pressure deviations from predefined target ranges.

According to institutional clinical management practice, invasive arterial blood pressure during off-pump coronary artery bypass grafting was maintained within a relatively stable range, with systolic blood pressure (SBP) targeted between 100 and 140 mmHg and diastolic blood pressure (DBP) between 60 and 90 mmHg. Blood pressure values exceeding these ranges were defined as intraoperative blood pressure abnormalities.

Based on blood pressure data recorded at fixed intervals in the anesthesia information management system, AUC values were calculated separately for systolic and diastolic blood pressure deviations beyond the predefined thresholds, including AUC for SBP < 100 mmHg (AUC_SBP < 100), SBP > 140 mmHg (AUC_SBP > 140), DBP < 60 mmHg (AUC_DBP < 60), and DBP > 90 mmHg (AUC_DBP > 90). AUC was calculated by integrating the absolute difference between the observed blood pressure values and the corresponding threshold over time, thereby reflecting both the severity and cumulative duration of blood pressure abnormalities. For regression analyses, AUC-based blood pressure exposure variables were rescaled to units of 10 mmHg·min to enhance interpretability of the corresponding odds ratios.

AUC_SBP < 100 (per 10 mmHg·min) and AUC_DBP < 60 (per 10 mmHg·min) represent cumulative hypotension burden but showed a zero-inflated distribution in which a substantial proportion of patients had no time below or above the pre-defined thresholds (i.e., AUC = 0). Directly presenting these variables as continuous medians would result in misleading values (e.g., 0 [0–0]) and obscure clinically important differences in those who experienced any hypotension burden. Therefore, these variables were separated into two components for descriptive purposes: a binary indicator of presence versus absence of hypotension burden (AUC = 0 vs. ≠0), and the distribution of AUC values among the subgroup with non-zero burden. This method provides clearer clinical interpretation, reduces the influence of zero-inflation, and ensures valid statistical comparisons using Chi-square and Mann–Whitney U tests.

To improve the interpretability of effect estimates in multivariable logistic regression models, all AUC variables were standardized per 10-mmHg units prior to analysis by dividing the original AUC values by 10. Accordingly, the odds ratios derived from regression analyses represent the change in risk of intraoperative high-volume diuresis associated with each additional 10-mmHg cumulative deviation from the target blood pressure range.

In contrast, other continuous variables (such as age, eGFR, or blood glucose) already possess inherent clinical units with intuitive scales. Their magnitudes do not require additional transformation, and presenting their effects in natural units maintains clarity without compromising interpretability.

### 2.5. Missing Data and Multiple Imputation

Given that some baseline and perioperative variables contained missing values and that the proportion of missing data for each variable was less than 10%, multiple imputation (MI) was applied to handle missing data in order to minimize potential bias associated with complete-case analysis. The extent of missing data for each variable is summarized in Supplementary Table S3. The imputation model included all clinical variables considered potentially related to the occurrence of intraoperative high-volume diuresis and study outcomes, including demographic characteristics, perioperative management variables, and hemodynamic parameters.

Continuous variables were imputed using predictive mean matching, whereas categorical variables were imputed using appropriate logistic or multinomial regression models. A total of 20 imputed datasets were generated, and regression coefficients and standard errors from each dataset were combined according to Rubin’s rules.

Multiple imputation was applied exclusively for multivariable logistic regression analyses to improve model stability and statistical efficiency. Descriptive analyses and baseline characteristic comparisons were performed using the original observed data. Variables used to define intraoperative high-volume diuresis, including intraoperative urine output, body weight, and surgical duration, were not included in the imputation model.

### 2.6. Statistical Analysis

Continuous variables were assessed for normality prior to analysis. Variables with a normal distribution are presented as mean ± standard deviation and were compared between groups using the independent-samples *t* test. Variables with a non-normal distribution are reported as median with interquartile range and were compared using the Mann–Whitney U test. Categorical variables are presented as counts and percentages and were compared using the Fisher test. Baseline characteristics were derived from complete-case analyses using the original dataset, without multiple imputation.

To identify factors independently associated with intraoperative high-volume diuresis, multivariable logistic regression analysis was conducted. Multiple imputation was applied exclusively for multivariable logistic regression analyses. Univariable screening was not used for variable selection to avoid excluding clinically relevant covariates based solely on statistical significance.

Multivariable models were constructed using a clinically informed block-wise approach without prior univariable screening, in order to avoid excluding potentially relevant predictors based solely on statistical significance. Guided by prior literature and routine clinical practice, variables with previously reported or clinically plausible associations with intraoperative urine output were entered in Block 1, whereas additional perioperative and hemodynamic variables were included in Block 2.

Formal collinearity diagnostics (e.g., variance inflation factors) were not performed. Instead, potential multicollinearity was addressed a priori through conceptual grouping of variables into predefined clinical domains and by avoiding simultaneous inclusion of closely overlapping measures. For variables with potential collinearity, such as baseline eGFR and serum creatinine, eGFR was selected for inclusion in Block 1 as the primary indicator of baseline renal function, while serum creatinine was not entered concurrently in the same block and was considered only in subsequent modeling steps (Block 2) to avoid redundancy.

Regression results are presented as odds ratios with corresponding 95% confidence intervals. Model discrimination was evaluated using receiver operating characteristic curve analysis, and the area under the curve was calculated.

All statistical analyses were performed using SPSS software (version 26.0; IBM Corp., Armonk, NY, USA). A two-sided *p* value < 0.05 was considered statistically significant.

## 3. Result

### 3.1. Study Population and Baseline Characteristics

A total of 1274 adult patients undergoing off-pump coronary artery bypass grafting (OPCABG) were included in the final analysis. According to the predefined criterion of intraoperative urine output (≥5 mL·kg^−1^·h^−1^), 505 patients (39.6%) were classified into the intraoperative high-volume diuresis group (diuresis group), while 769 patients (62.4%) constituted the normal urine output group (Table 1).

Patients in the diuresis group were significantly older than those in the normal urine output group (62.75 ± 7.96 vs. 60.99 ± 8.56 years, *p* < 0.001). They also had lower body weight (67.87 ± 9.75 vs. 74.98 ± 12.87 kg, *p* < 0.001) and shorter height (164.88 ± 7.53 vs. 166.85 ± 8.55 cm, *p* < 0.001).

The prevalence of hypertension was lower in the diuresis group compared with the normal group (55.0% vs. 65.5%, *p* < 0.001). Preoperative use of diuretics and angiotensin receptor blockers (ARB) was also less frequent among patients with intraoperative diuresis (10.9% vs. 17.0%, *p* = 0.002; and 10.9% vs. 14.8%, *p* = 0.043, respectively).

There were no significant differences between the two groups in sex distribution, presence of unstable angina pectoris, prior myocardial infarction, diabetes mellitus, heart failure, cerebral infarction, or valvular heart disease (all *p* > 0.05).

### 3.2. Intraoperative Characteristics and Hemodynamic Exposure

Intraoperative characteristics differed significantly between groups (Table 2). The diuresis group had a less proportion of patients with SBP < 100 mmHg (69.3% vs. 74.4%, *p* = 0.048), whereas the prevalence of DBP > 90 mmHg was similar between groups (*p* = 0.054). Cumulative hemodynamic exposure showed that AUC_SBP < 100 mmHg was significantly lower in the diuresis group (110.0 [40.0–246.23] vs. 135.0 [55.0–315.0] mmHg·min, *p* = 0.006), while AUC values for SBP > 140, DBP < 60, and DBP > 90 mmHg did not differ significantly.

The use of vasoactive agents was more common in the diuresis group, including dopamine (71.1% vs. 64.4%, *p* = 0.011) and norepinephrine (61.8% vs. 54.9%, *p* = 0.015). Intraoperative fluid administration was significantly greater in the diuresis group, with higher total fluid infusion volumes (median 2750 [2500–3250] mL vs. 2500 [2000–2750] mL, *p* < 0.001) and crystalloid infusion volumes (2000 [1500–2500] mL vs. 1700 [1250–2000] mL, *p* < 0.001). As expected, intraoperative urine output was markedly higher in the diuresis group (2200 [2000–2500] mL vs. 1200 [700–1600] mL, *p* < 0.001).

No significant differences were observed between groups in average heart rate, surgery duration, blood loss, or baseline levels of serum sodium, glucose, lactate, albumin, and B-type Natriuretic Peptide (BNP). Baseline renal function was slightly better in the diuresis group, with higher eGFR and lower serum creatinine and BUN levels (all *p* < 0.001).

### 3.3. Multivariable Logistic Regression Analysis

In the final multivariable logistic regression model (Block 2) (Table 3), several perioperative factors were independently associated with the occurrence of intraoperative high-volume diuresis.

Factors associated with an increased risk of intraoperative high-volume diuresis included older age (OR 1.073 per year, 95% CI 1.022–1.127; *p* = 0.005), greater total intraoperative fluid infusion after logarithmic transformation (OR 23.843, 95% CI 9.931–57.247; *p* < 0.001), and a history of hypertension (OR 1.682, 95% CI 1.284–2.203; *p* < 0.001).

In contrast, perioperative of diuretic use was independently associated with a lower likelihood of intraoperative high-volume diuresis (OR 0.586, 95% CI 0.389–0.881; *p* = 0.010). Baseline blood glucose also showed a modest inverse association with diuresis (OR 0.966 per 10 mg/dL increase, 95% CI 0.945–0.987; *p* = 0.002).

Among hemodynamic exposure variables standardized per 10 mmHg·min, cumulative exposure to systolic blood pressure below 100 mmHg was independently associated with a reduced risk of intraoperative high-volume diuresis (OR 0.989, 95% CI 0.983–0.995; *p* = 0.001). Cumulative exposure to systolic hypertension, diastolic hypotension, or diastolic hypertension was not significantly associated with the outcome.

BNP levels were also examined to explore the potential role of natriuretic peptides in intraoperative diuresis. However, BNP was not significantly associated with intraoperative high-volume diuresis.

Other variables, including diabetes, heart failure, myocardial infarction, cerebrovascular disease, mitral or aortic valve disease, baseline electrolyte levels, heart rate, vasoactive drug use, ARB use, crystalloid infusion volume, lactate, albumin, eGFR, Cr, BUN, and intraoperative blood loss, were not independently associated with intraoperative high-volume diuresis after adjustment.

### 3.4. Model Performance

**Figure 1:**
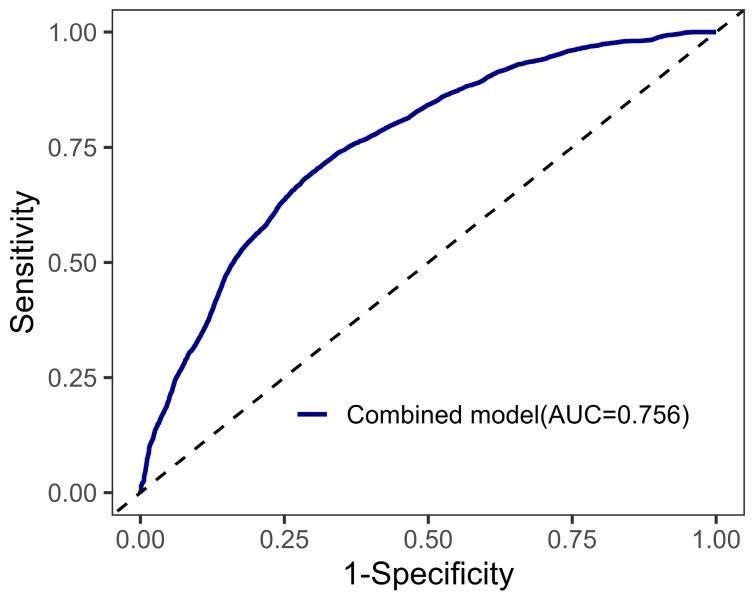
ROC curves for prediction of diuresis. ROC = Receiver Operating Characteristic; AUC = Area Under the Curve. The combined model includes age, hypertension, diuretics, LN_Totalfluidinfusion, T0 Glucose and AUC_SBP < 100. The dashed diagonal line represents the line of no discrimination (AUC = 0.5), indicating the performance of a non-informative test.

The discriminative performance of the final multivariable model was assessed using receiver operating characteristic curve analysis. The mean area under the curve across the 20 imputed datasets was 0.756, indicating acceptable discrimination for identifying patients at risk of intraoperative high-volume diuresis (Figure 1).

To further assess the robustness of the multivariable model, we performed additional sensitivity analyses. A multivariable model excluding total perioperative fluid infusion showed a reduced discriminative performance (AUC = 0.668; Supplementary Table S1), whereas an alternative model using fluid exposure indexed to operative time and body weight demonstrated improved discrimination (AUC = 0.803; Supplementary Table S2).

### 3.5. Postoperative Outcomes

Postoperative outcomes are summarized in Table 4. No significant differences were observed between groups in terms of postoperative mechanical ventilation duration, intensive care unit stay, or total hospital length of stay. However, patients in the diuresis group exhibited higher postoperative serum sodium levels (*p* < 0.001) and lower postoperative creatinine concentrations at both early (T2) and later (T3) postoperative time points (all *p* < 0.001).

Postoperative acute kidney injury (AKI) occurred in four patients overall (0.3%). The incidence of AKI did not differ significantly between the normal and diuresis groups (3/769 [0.4%] vs. 1/505 [0.2%], *p* = 0.653).

## 4. Discussion

This study focused on intraoperative high-volume diuresis, a common yet long-overlooked phenomenon during off-pump coronary artery bypass grafting. Using a large single-center cohort, we systematically examined its occurrence, perioperative associated factors, and clinical implications. Through multivariable modeling and quantitative hemodynamic assessment, we identified key predictors of intraoperative high-volume diuresis and explored the relationship between cumulative blood pressure exposure and urine output regulation, as well as the potential impact of this phenomenon on short-term postoperative outcomes. Collectively, these findings provide further insight into the clinical significance of intraoperative urine output changes during OPCABG and may inform more physiologically grounded perioperative management.

Based on these findings, the discussion below addresses preoperative risk stratification, intraoperative hemodynamic exposure, the clinical impact of intraoperative high-volume diuresis, and study limitations.

### 4.1. Preoperative Risk Factors Associated with Intraoperative High-Volume Diuresis

Multivariable analysis demonstrated that several preoperative characteristics were independently associated with intraoperative high-volume diuresis, indicating that perioperative urine output is partly influenced by baseline patient profiles. Increasing age and a history of hypertension were associated with an increased risk of intraoperative diuresis, whereas higher preoperative blood glucose levels were inversely associated with this outcome.

#### 4.1.1. Age

Age emerged as an independent risk factor for intraoperative high-volume diuresis, consistent with known age-related alterations in renal and neuroendocrine regulation. Aging is associated with progressive reductions in nephron number, diminished renal blood flow reserve, and impaired tubular handling of water and solutes [8,9]. In addition, alterations in antidiuretic hormone secretion and renal responsiveness have been described in older individuals. Although baseline vasopressin levels may be elevated, renal sensitivity to vasopressin-mediated water reabsorption appears attenuated with advancing age, potentially resulting in less stable water balance under conditions of perioperative stress [10,11]. These mechanisms may render elderly patients more susceptible to marked fluctuations in urine output during surgery. Surgical stress and hemodynamic perturbations during OPCABG may further amplify this vulnerability. This observation parallels age-related nocturia [12].

#### 4.1.2. Hypertension

Although hypertension appeared less frequent in the diuresis group in the unadjusted comparison, the association became positive after multivariable adjustment suggesting potential confounding by other perioperative factors. This finding indicates that the crude comparison may have been influenced by differences in perioperative fluid administration, baseline renal function or intraoperative hemodynamic exposure, which may mask the underlying relationship between hypertension and intraoperative urine output.

From a physiological perspective, hypertension may predispose patients to intraoperative polyuria through mechanisms related to both short-term hemodynamic responses and long-term renal structural changes. In patients with relatively early-stage hypertension or preserved renal function, elevated arterial pressure can enhance renal sodium and water excretion through the pressure natriuresis mechanism [13,14,15]. Increased renal perfusion pressure raises glomerular capillary pressure and glomerular filtration while reducing tubular sodium and water reabsorption, thereby promoting natriuresis and diuresis. During OPCABG, patients commonly receive fluid administration and experience fluctuations in arterial pressure, both of which may amplify this pressure-dependent excretory response. In the present study, patients in the diuresis group also had relatively higher preoperative eGFR, suggesting that preserved renal filtration capacity may facilitate increased urine output under perioperative volume loading, although baseline eGFR was not independently associated with diuresis in multivariable analysis.

In contrast, in patients with long-standing hypertension, persistent elevation of blood pressure may lead to renal arteriolar sclerosis and glomerular structural changes, commonly referred to as hypertensive nephrosclerosis [16,17]. These alterations may impair renal medullary perfusion and reduce tubular water reabsorption, leading to diminished urinary concentrating ability. Under such conditions, perioperative hemodynamic fluctuations and volume shifts may further accentuate urine output. Collectively, these mechanisms may contribute to the increased susceptibility to intraoperative polyuria observed in hypertensive patients undergoing OPCABG.

#### 4.1.3. Glucose

This study also found a mild inverse association between preoperative blood glucose levels and intraoperative high-volume diuresis, such that patients with higher preoperative glucose levels were slightly less likely to develop high-volume diuresis during surgery. However, the magnitude of this association was small, and its clinical and physiological interpretation should therefore be approached with caution. Given that this study did not concurrently collect indicators directly related to osmotic diuresis or antidiuretic hormone regulation, a definitive mechanistic explanation for this observation cannot be provided.

In general clinical settings, marked hyperglycemia may increase urine output through classical osmotic diuresis. However, in the present cohort, preoperative blood glucose levels were largely within a relatively normal range, and the distribution of a preoperative diabetes history did not differ significantly between patients with and without intraoperative high-volume diuresis. Therefore, it is unlikely that perioperative polyuria in OPCABG patients is primarily driven by elevated blood glucose through classical osmotic diuresis mechanisms. The mild inverse association observed in this study may instead reflect differences in baseline metabolic status, perioperative stress responses, or overall internal milieu regulation among patients, rather than a direct effect of blood glucose on urine output regulation.

Based on these considerations, the relationship between preoperative blood glucose levels and intraoperative high-volume diuresis is more appropriately interpreted as a statistical association rather than a physiological mechanism with clear causal implications, and its clinical impact appears to be limited.

#### 4.1.4. Body Size

In this study, intraoperative high-volume diuresis was defined using a weight-normalized urine output criterion (mL·kg^−1^·h^−1^). Therefore, body-size variables such as height and body weight were not included in the multivariable logistic regression model in order to avoid potential statistical correlation with the outcome definition itself. However, in the univariate comparison, patients in the diuresis group tended to have smaller body size, including lower body weight and shorter height.

This observation may partly relate to the manner in which perioperative fluid therapy is administered in clinical practice. Intraoperative fluid administration is generally adjusted according to hemodynamic conditions and surgical progress rather than being strictly normalized to body weight. As a result, patients with smaller body size may receive a similar absolute amount of fluid compared with larger patients, but this may represent a relatively greater effective volume load relative to their circulating blood volume. Because smaller individuals generally have a lower baseline circulating blood volume, such relative volume expansion may increase renal perfusion and glomerular filtration. Under these conditions, pressure-dependent diuresis may be more readily triggered during surgery. Taken together, differences in body size combined with perioperative fluid management patterns may partly explain the higher observed incidence of intraoperative high-volume diuresis among patients with smaller body size.

### 4.2. Effects of Perioperative Medications on Intraoperative Diuresis

Preoperative diuretic use was inversely associated with intraoperative high-volume diuresis. At our institution, diuretics are most commonly administered before surgery for the short-term management of heart failure or volume overload rather than as part of long-term maintenance therapy. Therefore, this association is more likely to reflect optimization of preoperative volume status rather than chronic renal adaptation to diuretics.

In patients with volume overload, elevated central venous pressure and atrial stretch may stimulate the release of natriuretic peptides and promote pressure-related diuresis. Preoperative diuretic therapy may help relieve venous congestion, reduce atrial pressure, and restore a more stable intravascular volume state before surgery. Under such conditions, the baseline drive for natriuretic or pressure-related diuresis during the intraoperative period may be attenuated. In addition, improved preoperative volume status may contribute to more stable perioperative hemodynamics and reduce abrupt shifts in renal perfusion or tubular sodium handling during surgery. As a result, patients receiving preoperative diuretics may exhibit a lower propensity for excessive urine output during OPCABG. Nevertheless, given the observational nature of the present study, these interpretations should be considered hypothesis-generating and require further investigation in prospective studies

Although previous studies have suggested that dopamine or norepinephrine may influence perioperative urine output by altering renal perfusion or intrarenal blood flow distribution, neither agent was independently associated with intraoperative high-volume diuresis in the present multivariable analysis [18,19,20]. This finding suggests that routine vasoactive drug use may not be a primary determinant of excessive intraoperative urine output.

### 4.3. Intraoperative Fluid Management and Hemodynamic Exposure

Our results indicate that intraoperative fluid management and hemodynamic exposure are closely associated with the occurrence of intraoperative high-volume diuresis. Patients who received a greater total volume of intraoperative fluids were more likely to develop excessive urine output during surgery. This observation suggests that larger fluid inputs may increase renal perfusion and filtration load, thereby facilitating pressure diuresis and volume-related fluid excretion, ultimately manifesting as markedly increased urine output. However, the association between intraoperative fluid administration and high-volume diuresis should be interpreted with caution. Given that the between-group difference in urine output substantially exceeded the difference in infused volume, a more plausible explanation is that the marked increase in urine output may have preceded and prompted additional fluid administration by anesthesiologists, rather than being solely caused by fluid loading. Therefore, intraoperative fluid infusion and urine output changes are more likely to reflect a dynamic, bidirectional interaction arising from real-time perioperative management, rather than a simple unidirectional causal relationship.

Given the intrinsic autoregulatory capacity of the kidney, intraoperative blood pressure status may play an important role in urine output regulation. In the present study, hemodynamic exposure was quantified using the area under the curve (AUC) to capture the cumulative burden of blood pressure deviations from predefined target ranges. We found that cumulative exposure to systolic blood pressure below 100 mmHg (AUC_SBP < 100) was inversely associated with the occurrence of intraoperative high-volume diuresis. This finding suggests that, compared with transient blood pressure fluctuations, sustained hemodynamic exposure patterns may better reflect the actual renal perfusion environment. During OPCABG, even modest reductions in systolic blood pressure, if persistent, may decrease renal perfusion pressure, attenuate pressure-induced diuresis, or activate neuroendocrine compensatory mechanisms favoring fluid retention, thereby reducing the risk of excessive intraoperative urine output [21]. Notably, the odds ratio for AUC_SBP < 100 (per 10 mmHg·min) was 0.989 indicating that its protective effect is attributable to the cumulative nature of hypotensive exposure rather than isolated or brief blood pressure decreases, underscoring the importance of sustained hemodynamic patterns in perioperative urine regulation.

In contrast, cumulative exposure to systolic blood pressure above 140 mmHg and abnormal diastolic blood pressure ranges were not independently associated with intraoperative high-volume diuresis, suggesting that not all blood pressure deviations exert equivalent effects on urine output regulation. Taken together, cumulative exposure to low-perfusion states may carry greater physiological relevance in perioperative renal fluid handling.

These findings may also help contextualize the observed association between pre-existing hypertension and intraoperative high-volume diuresis. While hypertension reflects chronic alterations in vascular and renal regulatory mechanisms, the occurrence of intraoperative diuresis ultimately depends on the real-time renal perfusion environment during surgery. Hypertensive patients may exhibit altered renal sodium handling and increased susceptibility to pressure-dependent diuresis under conditions of adequate or elevated perfusion pressure. However, when systolic blood pressure falls below the autoregulatory range for a sustained period, renal perfusion pressure may become insufficient to support pressure-driven diuresis, thereby attenuating urine output despite the underlying predisposition. In this context, baseline hypertensive status may increase susceptibility to intraoperative diuresis, whereas sustained low-perfusion states act as a counteracting factor that limits excessive urine output.

In addition to hemodynamic factors, neurohormonal regulation may also influence perioperative fluid balance. Natriuretic peptides may theoretically contribute to perioperative diuresis through their effects on sodium excretion and neurohormonal modulation. However, in our previous study, BNP was not identified as a major determinant of intraoperative urine output, and in the present analysis BNP was likewise not significantly associated with intraoperative high-volume diuresis.

### 4.4. Perioperative Impact of Intraoperative High-Volume Diuresis

From the perspective of perioperative outcomes, the present study further evaluated the impact of intraoperative high-volume diuresis on short-term clinical outcomes. The results showed that, under conditions of standardized monitoring and protocolized perioperative management, intraoperative high-volume diuresis was not associated with an increased incidence of acute kidney injury, nor was it associated with prolonged mechanical ventilation duration, ICU length of stay, or overall hospital stay. However, the overall incidence of AKI was very low, which may limit the statistical power to detect differences between groups. This low AKI incidence may reflect the relatively preserved baseline renal function of the study population and the exclusion of patients with severe perioperative circulatory instability.

Although patients in the diuresis group exhibited mildly elevated postoperative serum sodium levels, no concomitant abnormalities were observed in other metabolic indicators, including potassium, glucose, or lactate, and no clinically significant electrolyte disturbances or circulatory decompensation occurred. These findings suggest that, when appropriate supportive measures and dynamic interventions are provided, intraoperative high-volume diuresis itself does not translate into adverse short-term outcomes.

Nevertheless, perioperative management indicators indicate that intraoperative high-volume diuresis is not a phenomenon without management implications. Patients in the diuresis group required significantly greater intraoperative fluid administration and had a higher proportion of vasoactive drug use than those with normal urine output, suggesting that their circulatory status and volume balance were more prone to fluctuation and required more frequent and precise adjustments. In other words, although intraoperative high-volume diuresis did not directly lead to worse postoperative outcomes, it objectively increased the complexity and workload of perioperative management.

This observation indicates that, for anesthesiologists, intraoperative high-volume diuresis represents more of a management challenge than a direct pathological risk. The challenge lies in maintaining effective circulating blood volume, ensuring adequate perfusion of vital organs, and avoiding excessive fluid administration or hemodynamic instability while urine output increases substantially. Continuous dynamic assessment of volume status, hemodynamic trends, and electrolyte balance is therefore required to guide real-time decision-making. Importantly, the findings of this study suggest that, within an experienced perioperative team and a structured management framework, this challenge can be effectively controlled. Through timely fluid administration, appropriate use of vasoactive agents, and close monitoring of internal homeostasis, patients can maintain stable circulatory and metabolic conditions even in the presence of intraoperative high-volume diuresis. Therefore, intraoperative high-volume diuresis should not be interpreted as a direct signal of adverse outcomes, but rather as a phenomenon that tests the effectiveness of perioperative management and the understanding of underlying physiological mechanisms.

### 4.5. Clinical Implications

Several clinically relevant implications can be derived from the present findings. Intraoperative high-volume diuresis was a common phenomenon in patients undergoing OPCABG, affecting more than one-third of the cohort. It should therefore be recognized as a frequent perioperative occurrence rather than an exceptional event.

In addition, the identification of several preoperative predictors suggests that intraoperative high-volume diuresis may, to some extent, be anticipated before surgery. Awareness of patient-specific characteristics, such as advanced age, a history of hypertension, and preoperative medication use, may facilitate individualized perioperative planning and allow a more rational interpretation of intraoperative urine output fluctuations.

Finally, the findings of the present study may provide insights into the management of intraoperative high-volume diuresis. When excessive urine output occurs intraoperatively and the overall clinical condition permits, anesthesiologists may consider a comprehensive assessment of the hemodynamic environment, particularly factors influencing renal perfusion and urine output regulation. In our study, cumulative exposure to systolic blood pressure below 100 mmHg was associated with a lower likelihood of intraoperative high-volume diuresis, suggesting that renal perfusion pressure may play an important role in modulating pressure-dependent diuresis. However, this observation should not be interpreted as a recommendation to intentionally lower blood pressure, but rather as an indication that sustained hypotensive exposure may attenuate pressure-driven diuresis.

When pharmacological intervention is considered, the absence of an independent association between norepinephrine use and intraoperative high-volume diuresis suggests that routine vasoactive support may not effectively control excessive urine output. In contrast, modulation of antidiuretic pathways may represent a more targeted approach. Agents such as vasopressin or posterior pituitary hormone analogues may enhance antidiuretic effects and help stabilize urine output under appropriate clinical circumstances. These potential strategies should be interpreted cautiously and warrant further validation in prospective studies.

### 4.6. Limitations

This study has several limitations. First, as a single-center retrospective observational study, causal relationships cannot be established despite adjustment for potential confounders. In particular, intraoperative urine output and fluid administration are likely to interact bidirectionally in routine perioperative practice, and the temporal sequence as well as the direction of causality cannot be fully disentangled.

Given that total perioperative fluid infusion (CFI) showed a large effect size in the multivariable model (OR = 23.843), this finding indicates a strong association with intraoperative high-volume diuresis. However, increases in urine output frequently prompt reactive fluid administration during surgery; therefore, this association should be interpreted as correlational rather than causal. To further evaluate the influence of total fluid infusion on overall model performance and the stability of other predictors, we performed additional sensitivity analyses based on the available data. A multivariable model excluding total fluid infusion demonstrated a reduced discriminative performance (AUC = 0.688), indicating that fluid infusion contributes substantially to predictive performance. In addition, when fluid exposure was expressed using time- or body-weight-adjusted indices, model discrimination improved (AUC = 0.805). Importantly, across all model specifications, the direction of the main predictors—including age, hypertension history, preoperative diuretic and cumulative systolic hypotension exposure—remained consistent. These findings suggest that fluid-related variables are strong predictors of intraoperative high-volume diuresis and that their mode of expression influences model discrimination; however, prediction of intraoperative diuresis does not rely on a single formulation of fluid exposure, and different expressions of fluid variables affect predictive performance without altering the underlying association structure.

Second, this study assumed a linear relationship between age, renal function, and the outcome, but did not further explore potential threshold effects or nonlinear associations, which may have provided additional insights into the risk profile of intraoperative high-volume diuresis.

Third, vasoactive medication exposure was analyzed as a binary variable without detailed dosing information, precluding assessment of potential dose–response relationships.

Finally, patients requiring intraoperative intra-aortic balloon pump support or conversion to cardiopulmonary bypass were excluded. As a result, the findings may primarily apply to relatively hemodynamically stable OPCABG patients and may not be fully generalizable to higher-risk surgical populations.

## 5. Conclusions

Intraoperative high-volume diuresis is a common phenomenon in patients undergoing off-pump coronary artery bypass grafting and is associated with a combination of baseline patient characteristics, preoperative medication management, and perioperative fluid and hemodynamic patterns. The risk of excessive intraoperative urine output can be partially anticipated before and during surgery, allowing for a more informed interpretation of intraoperative urine output changes.

Together with our previous studies on perioperative hormonal responses [7], the present findings support the concept that intraoperative high-volume diuresis during OPCABG represents a mixed physiological process involving both hemodynamic influences and relative neuroendocrine dysregulation, rather than a purely volume-driven mechanism. The observed associations with baseline renal characteristics and cumulative blood pressure exposure further reinforce this integrated pathophysiological framework.

Recognition of these associated factors may facilitate more nuanced, individualized, and physiologically informed perioperative management strategies. From a translational perspective, modulation of the antidiuretic axis may represent a potential direction for future investigation; however, any interventional approaches should be interpreted cautiously and require prospective validation before clinical application.

## Figures and Tables

**Table 1:** Baseline characteristics between normal and diuresis groups.

Variable	Normal (*n* = 769)	Diuresis (*n* = 505)	Mean Difference (95% CI)	*χ* ^2^ */t*	*p* Value
Male, *n* (%)	604 (78.5)	378 (74.9)		2.352	0.125
Weight (kg)	74.98 ± 12.87	67.87 ± 9.75	7.11 (5.80, 8.43)	10.59	<0.001
Height (cm)	166.85 ± 8.55	164.88 ± 7.53	1.97 (1.06, 2.89)	4.22	<0.001
Age (years)	60.99 ± 8.56	62.75 ± 7.96	−1.176 (−2.70–0.83)	−3.70	<0.001
Angina, *n* (%)	730 (94.9)	487 (96.4)		1.620	0.203
Myocardial infarction, *n* (%)	37 (4.8)	15 (3.0)		2.639	0.104
Hypertension, *n* (%)	504 (65.5)	278 (55.0)		14.151	<0.001
Diabetes, *n* (%)	320 (41.6)	199 (39.4)		0.615	0.433
Heart failure, *n* (%)	15 (2.0)	6 (1.2)		1.093	0.296
Cerebral infarction, *n* (%)	104 (13.5)	73 (14.5)		0.211	0.638
Mitral valvular disease, *n* (%)	46 (6.0)	25 (5.0)		0.616	0.433
Tricuspid valve disease, *n* (%)	33 (4.3)	27 (5.3)		0.756	0.384
Aortic valve disease, *n* (%)	20 (2.6)	10 (2.0)		0.511	0.475
Diuretic, *n* (%)	131 (17.0)	55 (10.9)		9.229	0.002
ARB, *n* (%)	114 (14.8)	55 (10.9)		4.099	0.043

Baseline characteristics are presented based on complete-case analysis. Data are presented as mean ± SD or *n* (%). Statistical comparisons were performed using an independent *t* test for normally distributed continuous variables, and the *χ*^2^ test for categorical variables. MI = Myocardial infarction, ARB = Angiotensin II receptor blocker.

**Table 2:** Intraoperative variables between normal and diuresis groups.

Variable	Normal (*n* = 769)	Diuresis (*n* = 505)	*χ* ^2^ */Z*	*p* Value
SBP < 100 AUC ≠ 0, *n* (%)	572 (74.4)	350 (69.3)	3.927	0.048
DBP > 90 AUC ≠ 0, *n* (%)	230 (29.9)	126 (25.0)	3.722	0.054
AUC_SBP < 100 among AUC ≠ 0 (per mmHg·min)	135.0 [55.0–315.0]	110.0 [40.0–246.23]	−2.770	0.006
AUC_DBP > 90 among AUC ≠ 0 (per mmHg·min)	32.5 [15.0–90.0]	32.5 [15.0–66.25]	−0.714	0.475
AUC_SBP > 140 (per mmHg·min)	395.0 [125.0–870.0]	385.0 [160.0–775.0]	−0.583	0.560
AUC_DBP < 60 (per mmHg·min)	200.0 [50.0–485.0]	155.0 [40.0–470.0]	−0.570	0.569
Dopamine, *n* (%)	494 (64.2)	359 (71.1)	6.464	0.011
NE, *n* (%)	422 (54.9)	312 (61.8)	5.953	0.015
Average HR (Beats/min)	67.5 [61.6–73.6]	68.3 [62.4–74.7]	−1.275	0.202
Surgery time (hours)	5.1 [4.5–5.7]	5.1 [4.6–5.6]	−0.825	0.409
Total fluid infusion (mL)	2500.0 [2000.0–2750.0]	2750.0 [2500.0–3250.0]	−10.946	<0.001
Crystal fluid infusion (mL)	1700.0 [1250.0–2000.0]	2000.0 [1500.0–2500.0]	−7.885	<0.001
T0 Sodium (mmol/L)	139.0 [137.0–141.0]	139.0 [137.0–140.0]	−0.951	0.341
T0 Potassium (mmol/L)	3.80 [3.60–4.00]	3.80 [3.60–4.00]	−2.209	0.027
T0 Glucose (mg/dL)	169.0 [134.5–229.5]	163.5 [133.0–208.0]	−1.272	0.203
T0 Lactate (mmol/L)	1.8 [1.4–2.2]	1.8 [1.4–2.2]	−1.124	0.261
T0 Albumin (g/dL)	41.3 [39.2–43.7]	41.0 [38.8–43.7]	−1.192	0.233
T0 eGFR (mL/min·1.73 m^2^)	92.8 [81.0–100.2]	94.4 [88.8–100.6]	−3.319	<0.001
T0 Cr (µmol/L)	72.0 [62.5–84.2]	67.2 [59.5–75.5]	−5.842	<0.001
T0 BUN (mmol/L)	6.2 [5.2–7.4]	5.9 [4.9–7.0]	−3.626	<0.001
T0 BNP (pg/mL)	84.4 [43.4–187.3]	72.2 [39.9–144.3]	−1.562	0.127
Blood Loss (mL)	600.0 [500.0–700.0]	600.0 [500.0–700.0]	−0.160	0.872
Urine (mL)	1200.0 [700.0–1600.0]	2200.0 [2000.0–2500.0]	−25.053	<0.001

Baseline characteristics are presented based on complete-case analysis. Data are presented as mean ± SD, median [IQR], or *n* (%). Statistical comparisons were performed using an independent *t* test for normally distributed continuous variables, the Mann–Whitney U test for non-normally distributed continuous variables, and the χ^2^ test for categorical variables. AUC_SBP = Area under the curve of systolic blood pressure, AUC_DBP = Area under the curve of diastolic blood pressure, NE = Norepinephrine, HR = Heart beats, eGFR = Estimated glomerular filtration rate, Cr = Serum creatinine, BUN = Blood urea nitrogen, BNP = B-type Natriuretic Peptide.

**Table 3:** Final multivariable logistic regression model (Block 2) identifying independent risk factors for intraoperative high-volume diuresis.

Variable	B	*p*-Value	OR	95%CI for OR
Sex (1 = male)	−0.227	0.569	0758	0.292–1.967
Age (years)	0.070	0.005	1.073	1.022–1.127
Dopamine (1)	0.095	0.503	1.100	0.832–1.453
NE (1)	0.070	0.599	1.073	0.825–1.395
Hypertension (1)	0.520	<0.001	1.682	1.284–2.203
Diabetes (1)	−0.115	0.445	0.892	0.665–1.196
Heart failure (1)	−0.114	0.847	0.893	0.282–2.823
Diuretic (1)	−0.535	0.010	0.586	0.389–0.881
LN_Totalfluidinfusion	3.171	<0.001	23.843	9.931–57.247
T0 Potassium (mmol/L)	−0.350	0.153	0.705	0.436–1.139
T0 Sodium (mmol/L)	−0.049	0.080	0.952	0.901–1.006
T0 Glucose (per 10 mg/dL)	−0.035	0.002	0.966	0.945–0.987
T0 eGFR (mL/min·1.73 m^2^)	0.037	0.301	1.037	0.968–1.112
AUC_SBP > 140 (per 10 mmHg·min)	0.000	0.949	1.000	0.998–1.002
AUC_SBP < 100 (per 10 mmHg·min)	−0.011	0.001	0.989	0.983–0.995
AUC_DBP > 90 (per 10 mmHg·min)	−0.008	0.351	0.992	0.976–1.009
AUC_DBP < 60 (per 10 mmHg·min)	0.001	0.524	1.001	0.999–1.003
BNP (mg/dL)	0.000	0.596	1.000	0.999–1.001
HR (Beats/min)	0.013	0.094	1.014	0.998–1.030
Angina, (1)	1.112	0.246	3.040	0.464–19.901
Myocardial infarction, (1)	1.538	0.132	4.653	0.631–34.326
Cerebral infarction, (1)	−0.202	0.279	0.821	0.570–1.184
Mitral valvular disease, (1)	0.314	0.452	1.369	0.604–3.105
Tricuspid valve disease, (1)	−0.660	0.135	0.517	0.217–1.229
Aortic valve disease, (1)	0.586	0.268	1.797	0.637–5.069
T0 Lactate (mmol/L)	−0.013	0.894	0.987	0.810–1.202
T0 Albumin (g/dL)	−0.002	0.910	0.998	0.959–1.038
T0 Cr (µmol/L)	0.006	0.845	1.006	0.949–1.065
T0 BUN (mmol/L)	−0.064	0.116	0.938	0.865–1.016
ARB (1)	0.040	0.853	1.041	0.683–1.587
LN_Crystalfluidinfusion	−0.339	0.280	0.713	0.386–1.317

SubSubsec: 1 = yes, B = regression coefficient; OR = odds ratio; CI = confidence interval; OR < 1 indicates that an increase in the variable is associated with a decreased likelihood of diuresis.

**Table 4:** Postoperative outcomes between normal and diuresis groups.

Variable	Normal (*n* = 769)	Diuresis (*n* = 505)	Z Value/χ^2^	*p* Value	H-L Median Difference (95%CI)
Blood Loss (mL)	600 [500–600]	600 [500–600]	−0.160	0.872	0.000 (0.000–0.000)
Ventilation (hours)	19.0 [16.0–24.0]	19.0 [15.0–23.0]	−0.102	0.919	0.000 (−1.000–1.000)
ICU stay (days)	0.96 [0.81–1.84]	0.91 [0.79–1.72]	−0.899	0.369	0.020 (−0.020–0.050)
Hospital stay (days)	15.0 [12.0–19.0]	15.0 [13.0–18.0]	−0.786	0.432	0.000 (−1.000–0.000)
T1 Sodium (mmol/L)	144.0 [142.0–146.0]	147.0 [145.0–149.0]	−12.370	<0.001	−3.000 (−3.000–−2.000)
T1 Potassium (mmol/L)	4.10 [3.90–4.40]	4.20 [4.00–4.40]	−1.283	0.200	0.000 (−0.100–0.000)
T1 Glucose (mg/dL)	144.0 [120.6–174.6]	145.8 [124.2–176.4]	−1.284	0.199	−0.200 (−0.400–0.100)
T1 Lactate (mmol/L)	0.70 [0.60–1.00]	0.70 [0.60–0.90]	−0.303	0.762	0.000 (0.000–0.000)
T2 Cr (µmol/L)	68.90 [57.00–80.20]	61.20 [52.90–72.20]	−6.707	<0.001	6.400 (4.600–8.200)
T3 Cr (µmol/L)	64.10 [54.30–77.80]	59.70 [51.30–70.20]	−5.137	<0.001	4.700 (2.900–6.500)
AKI, *n* (%)	3 (0.4%)	1 (0.2%)		0.653	

Data are presented as mean ± SD, median [IQR], or *n* (%). Statistical comparisons were performed using an independent *t* test for normally distributed continuous variables, the Mann–Whitney U test for non-normally distributed continuous variables, and the Fisher exact test for categorical variables. ICU = Intensive care unit, AKI = Acute kidney injury. H-L = Hodges–Lehmann.

## Data Availability

The datasets generated and analyzed during the current study are deposited in the Zenodo repository and are available at https://doi.org/10.5281/zenodo.18277223. Access to the data is restricted due to ethical and privacy considerations. The datasets can be made available from the corresponding author upon reasonable request and with appropriate institutional approval.

## Supplementary Materials

The following supporting information can be downloaded at: https://www.mdpi.com/article/10.3390/jcm15062331/s1, Table S1. Multi-variable model excluding total perioperative fluid infusion; Table S2. alternative model using fluid exposure indexed to operative time and body weight; Table S3. Missing data and multiple imputation models.

## Abbreviations

The following abbreviations are used in this manuscript:

AKI	Acute Kidney Injury
ARB	Angiotensin II Receptor Blocker
AUC	Area Under the Curve
BP	Blood Pressure
BUN	Blood Urea Nitrogen
CFI	Crystal Fluid Infusion
CI	Confidence Interval
Cr	Creatinine
DBP	Diastolic Blood Pressure
eGFR	Estimated Glomerular Filtration Rate
HR	Heart Rate
ICU	Intensive Care Unit
KDIGO	Kidney Disease Improving Global Outcomes
MI	Multiple Imputation
NE	Norepinephrine
OPCABG	Off-pump Coronary Artery Bypass Grafting
OR	Odds Ratio
ROC	Receiver Operating Characteristic
RR	Risk Ratio
SBP	Systolic Blood Pressure
SD	Standard Deviation
U test	Mann–Whitney U test

## References

[1] Fountas A., Coulden A., Fernández-García S., Tsermoulas G., Allotey J., Karavitaki N. (2024). Central diabetes insipidus (vasopressin deficiency) after surgery for pituitary tumours: A systematic review and meta-analysis. Eur. J. Endocrinol..

[2] Li J., Zhang Z. (2024). Establishment and validation of a predictive nomogram for polyuria during general anesthesia in thoracic surgery. J. Cardiothorac. Surg..

[3] Zhou S., Tian Z., Chu T., Yu S., Xin Y., Xu A. (2025). Analysis of factors associated with polyuria in spinal surgery: A retrospective study. BMC Anesthesiol..

[4] Wang C., Jiang Y., Jiang X., Chen S. (2021). On-pump beating heart versus conventional on-pump coronary artery bypass grafting on clinical outcomes: A meta-analysis. J. Thorac. Dis..

[5] Karabdic I.H., Strauss S., Granov N., Hadzimehmedagic A., Kabil E., Djedovi M., Kurtagic D., Berberovic B. (2023). Off pump Versus On pump Coronary Artery Bypass Grafting: Short-term Outcomes. Acta Inform. Med..

[6] Shim J.K., Kim K.-S., Couture P., Denault A., Kwak Y.-L., Yoo K.-J., Youn Y.-N. (2023). Hemodynamic management during off-pump coronary artery bypass surgery: A narrative review of proper targets for safe execution and troubleshooting. Korean J. Anesthesiol..

[7] Hou Y., Li S., Cai F., Luo F., Ma J. (2025). Hormonal and Osmoregulatory Responses in Intraoperative High-Volume Diuresis During Off-Pump Coronary Artery Bypass Grafting: An Exploratory Cohort Study. J. Clin. Med..

[8] Sands J.M. (2012). Urine concentrating and diluting ability during aging. J. Gerontol. A Biol. Sci. Med. Sci..

[9] Luckey A.E., Parsa C.J. (2003). Fluid and electrolytes in the aged. Arch. Surg..

[10] Mutig K., Lebedeva S., Singh P.B. (2025). Inflammation and vasopressin hypersecretion in aging. Front. Endocrinol..

[11] Tian Y., Serino R., Verbalis J.G. (2004). Downregulation of renal vasopressin V2 receptor and aquaporin-2 expression parallels age-associated defects in urine concentration. Am. J. Physiol. Renal Physiol..

[12] Asplund R., Aberg H. (1991). Diurnal variation in the levels of antidiuretic hormone in the elderly. J. Intern. Med..

[13] Kuwahara K. (2021). The natriuretic peptide system in heart failure: Diagnostic and therapeutic implications. Pharmacol. Ther..

[14] Ivy J.R., Bailey M.A. (2014). Pressure natriuresis and the renal control of arterial blood pressure. J. Physiol..

[15] Granger J.P., Alexander B.T., Llinas M. (2002). Mechanisms of pressure natriuresis. Curr. Hypertens. Rep..

[16] Hill G.S. (2008). Hypertensive nephrosclerosis. Curr. Opin. Nephrol. Hypertens..

[17] Agaba E.I., Rohrscheib M., Tzamaloukas A.H. (2012). The renal concentrating mechanism and the clinical consequences of its loss. Niger. Med. J..

[18] Bellomo R., Chapman M., Finfer S., Hickling K., Myburgh J. (2000). Low-dose dopamine in patients with early renal dysfunction: A placebo-controlled randomised trial. Australian and New Zealand Intensive Care Society (ANZICS) Clinical Trials Group. Lancet.

[19] de Werra P., Bracco D. (1999). Dopamine à dose rénale: Mythe ou réalité? [Renal-protective dose of dopamine: Myth or reality?]. Rev. Med. Suisse Romande.

[20] Redfors B., Bragadottir G., Sellgren J., Swärd K., Ricksten S.E. (2011). Effects of norepinephrine on renal perfusion, filtration and oxygenation in vasodilatory shock and acute kidney injury. Intensive Care Med..

[21] Baek E.J., Kim S. (2021). Current Understanding of Pressure Natriuresis. Electrolyte Blood Press..

