# Supplementary material for: Intraoperative High-Volume Diuresis During Off-Pump Coronary Artery Bypass Grafting: Risk Factors and Clinical Impact
Source: J Clin Med. 2026 Mar 18;15(6):2331. doi: 10.3390/jcm15062331 (PMC13026217; doi:10.3390/jcm15062331)
Supplement: Supplementary file 1 [file jcm-15-02331-s001.zip › jcm-4176209-supplementary.pdf]

Supplementary Materials

Supplementary Table S1. Multi-variable model excluding total perioperative fluid infusion

Variable	B	*p*-Value	OR	95%CI for OR
Sex（1=male）	-0.112	0.980	0.805	0.366-2.182
Age (years)	0.061	0.009	1.063	1.015-1.112
Dopamine (1)	0.192	0.155	1.212	0.930-1.579
NE (1)	0.193	0.129	1.213	0.945-1.556
Hypertension (1)	0.483	<0.001	1.621	1.254-2.096
Diabetes (1)	-0.086	0.545	0.917	0.694-1.213
Heart failure (1)	-0.124	0.821	0.883	0.300-2.598
Diuretic (1)	-0.519	0.009	0.595	0.404-0.876
T0 Potassium(mmol/L)	-0.414	0.081	0.661	0.416-1.052
T0 Sodium(mmol/L)	-0.052	0.053	0.949	0.900-1.001
T0 Glucose（per 10 mg/dL)	-0.033	0.002	0.968	0.948–0.988
T0 eGFR (mL/min·1.73 m2)	0.032	0.340	1.032	0.967-1.102
AUC_SBP>140 (per 10 mmHg·min)	0.000	0.819	1.000	0.998-1.002
AUC_SBP<100 (per 10 mmHg·min	-0.008	0.005	0.992	0.986-0.997
AUC_DBP>90 (per 10 mmHg·min)	-0.011	0.150	0.989	0.974-1.004
AUC_DBP<60 (per 10 mmHg·min)	0.001	0.467	1.001	0.999-1.003
BNP(mg/mL)	0.000	0.609	1.000	0.999-1.001
HR (Beats/min)	0.028	<0.001	1.029	1.013-1.044
Angina, (1)	0.858	0.376	2.358	0.352-15.780
Myocardial infarction, (1)	1.288	0.208	3.625	0.489-26.888
Cerebral infarction, (1)	-0.208	0.247	0.812	0.571-1.155
Mitral valvular disease, (1)	0.694	0.082	2.003	0.915-4.383
Tricuspid valve disease, (1)	-0.987	0.020	0.373	0.162-0.858
Aortic valve disease, (1)	0.468	0.348	1.596	0.601-4.240
T0 Lactate(mmol/L)	0.006	0.954	1.006	0.829-1.220
T0 Albumin (g/dL)	0.004	0.840	1.004	0.968-1.041
T0 Cr (μmol/L)	0.002	0.932	1.002	0.950-1.058
T0 BUN (mmol/L)	-0.077	0.049	0.926	0.858-1.000
ARB(1)	0.000	0.998	1.000	0.669-1.495

This supplementary table presents a sensitivity analysis using a multivariable regression model that excluded total perioperative fluid infusion. Model specification, covariate selection, and variable scaling were otherwise identical to those of the primary multivariable model. AUC-based blood pressure exposure variables were scaled per 10 mmHg·min.

Supplementary Table S2. Alternative model using fluid exposure indexed to operative time and body weight.

Variable	B	*p*-Value	OR	95%CI for OR
Sex（1=male）	0.104	0.839	1.110	0.406-3.036
Age (years)	0.060	0.024	1.062	1.008-1.118
Dopamine (1)	0.204	0.172	1.226	0.915-1.644
NE (1)	0.160	0.253	1.174	0.892-1.545
Hypertension (1)	0.371	0.010	1.450	1.092-1.925
Diabetes (1)	-0.247	0.117	0.781	0.573-1.064
Heart failure (1)	-0.110	0.859	0.896	0.267-3.008
Diuretic (1)	-0.533	0.015	0.587	0.381-0.903
Totalfluidinfusion/Time ·Weight	0.571	<0.001	1.770	1.562-2.006
T0 Potassium(mmol/L)	-0.519	0.042	0.595	0.361-0.982
T0 Sodium(mmol/L)	-0.052	0.082	0.949	0.895-1.007
T0 Glucose（per 10 mg/dL)	-0.035	0.002	0.965	0.944–0.987

T0 eGFR (mL/min·1.73 m2)	0.061	0.106	1.063	0.987-1.144
AUC_SBP>140 (per 10 mmHg·min)	0.001	0.481	1.001	0.999-1.003
AUC_SBP<100 (per 10 mmHg·min)	-0.009	0.005	0.991	0.984-0.997
AUC_DBP>90 (per 10 mmHg·min)	-0.005	0.566	0.995	0.979-1.011
AUC_DBP<60 (per 10 mmHg·min)	0.000	0.740	1.000	0.998-1.003
BNP(mg/dL)	-0.001	0.155	0.999	0.998-1.000
HR (Beats/min)	0.008	0.328	1.008	0.992-1.025
Angina, (1)	0.801	0.408	2.228	0.334-14.883
Myocardial infarction, (1)	1.141	0.270	3.130	0.412-23.790
Cerebral infarction, (1)	-0.137	0489	0.872	0.592-1.285
Mitral valvular disease, (1)	0.382	0.378	1.465	0.627-3.423
Tricuspid valve disease, (1)	-0.752	0.099	0.471	0.193-1.153
Aortic valve disease, (1)	0.467	0.398	1.595	0.540-4.714
T0 Lactate(mmol/L)	0.039	0.714	1.040	0.845-1.279
T0 Albumin (g/dL)	0.001	0.943	1.001	0.961-1.044
T0 Cr (μmol/L)	0.029	0.344	1.030	0.969-1.094
T0 BUN (mmol/L)	-0.098	0.023	0.906	0.832-0.987
ARB(1)	0.006	0.979	1.006	0.646-1.567
Crystalfluidinfusion/Time ·Weight	-0.092	0.159	0.912	0.803-1.037

Supplementary table presents an alternative multivariable model in which fluid exposure was indexed to operative time and body weight. All other covariates, modeling strategy, and scaling of AUC-based blood pressure exposure variables (per 10 mmHg·min) were consistent with those of the primary model.

Supplementary Table S3. Missing data and multiple imputation models

Variable	Imputation model	Missing values,n(%)	Number of imputations
T0 Albumin	Logistic regression	16（≈1.3%）	20
T0 BUN	Logistic regression	16（≈1.3%）	20
T0eGFR	Logistic regression	20（≈1.3%）	20
T0 Cr	Logistic regression	62（≈1.6%）	20
AUC_SBP<100	Logistic regression	72（≈5.7%）	20
AUC_SBP>140	Logistic regression	72（≈5.7%）	20
AUC_DBP<60	Logistic regression	72（≈5.7%）	20
AUC_DBP>90	Logistic regression	72（≈5.7%）	20
T0 Sodium	Logistic regression	96（≈7.5%）	20
T0 Potassium	Logistic regression	96（≈7.5%）	20
T0 Glucose	Logistic regression	96（≈7.5%）	20
T0 Lactate	Logistic regression	96（≈7.5%）	20
T0 BNP	Logistic regression	318（≈25%）	20
